# Brush-paintable and highly stretchable Ag nanowire and PEDOT:PSS hybrid electrodes

**DOI:** 10.1038/s41598-017-14951-3

**Published:** 2017-10-31

**Authors:** Ji-Eun Lim, Sang-Mok Lee, Seok-Soon Kim, Tae-Woong Kim, Hyun-Woo Koo, Han-Ki Kim

**Affiliations:** 1 Department of Advanced Materials Engineering for Information and Electronics, Kyung-Hee University, 1 Seochen-dong, Yongin-si, Gyeonggi-do, Seoul, 446-701 Republic of Korea; 2 Department of Nano and Chemical Engineering, Kunsan National University, Kunsan, Jeollabuk-do 753, Seoul, Korea; 3 OLED R&D Center, Samsung Display, 230 Nongseo-dong, Gyeonggi-do, Yongin-si, 17113 Republic of Korea

## Abstract

Highly transparent and stretchable Ag nanowire (NW)/poly(3,4-ethylenedioxythiophene):poly(styrenesulfonate) (PEDOT:PSS) hybrid electrodes were prepared on stretchable polyurethane substrates by using simple and cost-effective brush painting technique. The optimized Ag NW/PEDOT:PSS hybrid electrode showed a sheet resistance of 19.7 Ohm/square and a high optical transmittance of 88.64% comparable to conventional ITO electrode. It was found that shear stress of the paintbrush led to an effective lateral alignment of the Ag NWs into the PEDOT:PSS matrix during brush painting process. In addition, we investigated mechanical properties of the brush painted Ag NW/PEDOT:PSS hybrid electrode using inner/outer bending test, stretching tests, twisting test and rolling test in detail. The optimized brush painted Ag NW/PEDOT:PSS electrode showed a higher strain (~30%) than brush painted Ag NW or sputtered ITO electrode. Furthermore, we demonstrated the outstanding stretchability of brush painted Ag NW/PEDOT:PSS hybrid electrode in two applications: stretchable interconnectors and stretchable electrodes for stretchable and wearable thin film heaters. These results provide clear evidence for its potential and widespread applications in next-generation, stretchable displays, solar cells, and electronic devices.

## Introduction

Stretchable and wearable electronics such as electronic skin, human-machine interfaces, and health monitoring sensors have attracted great attention and are indicative of the future of the electronics industry^1–10^. The development of soft and highly stretchable electrodes or interconnectors prepared by a cost-efficient simple process is critical for stretchable and wearable electronics because the mechanical performance or reliability of stretchable devices is affected by the electrical, optical, and mechanical properties of electrodes^11–15^. To fabricate stretchable electrodes or interconnectors, conducting polymers or inorganic solutions have been coated on intrinsically stretchable substrates or wavy/buckled substrates with specific shapes (zigzag or horseshoe) by several printing processes or conventional photolithography processes^16–20^. Horseshoe-shaped opaque metal electrodes, printed poly(3,4-ethylene dioxylene thiophene):poly (styrene sulfonic acid) (PEDOT:PSS), printed carbon nanotubes (CNTs), printed carbon composites, transferred graphene, and printed Ag nanowires coated on stretchable polydimethylsiloxane (PDMS) substrates have been widely used as stretchable electrodes for stretchable and wearable electronics^7–10,21–23^. In particular, Ag nanowire-based stretchable electrodes or interconnectors prepared by conventional spin coating, inkjet printing, transfer printing, drop casting, air-spray coating, and Meyer rod coating on flat or wavy patterned PDMS substrates have been extensively investigated^24–27^. Brush painting is a simple and cost-efficient solution process that has been extensively reported due to its advantages, which include low material loss, ultra-low-cost and vacuum-free processing, high manufacturability, and excellent compatibility with typical roll-to-roll processing.

Brush-painting can compete with other printing techniques in the area of functional devices as well as low-cost applications because it has the following advantages: control of film thickness from ~10 nm to a few hundred nanometers (due to fast solidification of films on a hot-substrate), shear stress-induced ordering of polymer chains or 1-dimensional nanostructures caused by contact with the brush, and processability on various flat and textured substrates. Reports of the successful operation of brush-painted organic thin film transistors and brush-painted perovskite solar cells have been published since Kim *et al*. reported the feasibility of brush-painted polymer solar cells^28–31^. We previously reported electrodes made of brush-painted Ag nanowires, CNTs, PEDOT:PSS, Sn-doped In_2_O_3_ nanoparticles, and Ti-doped In_2_O_3_ nanoparticles for paintable organic solar cells^32–37^. Using various transparent conducting electrode inks, we obtained low resistance, highly transparent electrodes by a simple brush process under ambient conditions. Although several types of brush-painted transparent electrodes have been reported, detailed investigation of stretchable transparent electrode fabricated by simple brush painted on stretchable thermoplastic substrate.

In our previous work, we introduce the PEDOT:PSS/Ag NW/PEDOT:PSS multilayer with a sheet resistance of 13.96 Ohm/square and transmittance of 80.48% using simple brush process^34^. However, the multi-brush process to fabricate hybrid electrodes led to ununiform coating and irregular distribution of the Ag NW in PEDOT:PSS matrix. Although successful demonstration of devices based on brush-painted transparent electrodes have been reported, there have been no reports on brush-painted electrodes for stretchable and multifunctional devices. Therefore, we introduced excellent stretchable Ag NW/PEDOT:PSS hybrid electrodes prepared by direct writing and painting of Ag NW and PEDOT:PSS mixed ink on a stretchable polyurethane (PU) substrate. We used the PU substrate as an alternative to conventional PDMS substrates due to its high stretchability, high abrasion resistance, high impact resistance, and large-area scalability^3,38^. To our knowledge, this is the first demonstration of stretchable Ag NW/PEDOT:PSS hybrid electrodes on a PU substrate by simple brush-painting. We produced writable and paintable electrodes with low sheet resistance, high optical transmittance, and high stretchability by direct brushing of Ag NW and PEDOT:PSS mixed ink on the stretchable PU substrate. We also compared the properties of brush-painted Ag NWs electrodes and brush-painted Ag NW/PEDOT:PSS hybrid electrodes to show the effect of the conducting PEDOT:PSS matrix. Furthermore, we demonstrated the outstanding stretchability of Ag NW/PEDOT:PSS electrodes in two applications: stretchable interconnectors and stretchable electrodes for stretchable and wearable thin film heaters. These results clearly show the potential of these materials for use in wide-spread applications in next-generation, cost-efficient paintable and stretchable electronics.

## Results

**Figure 1:**
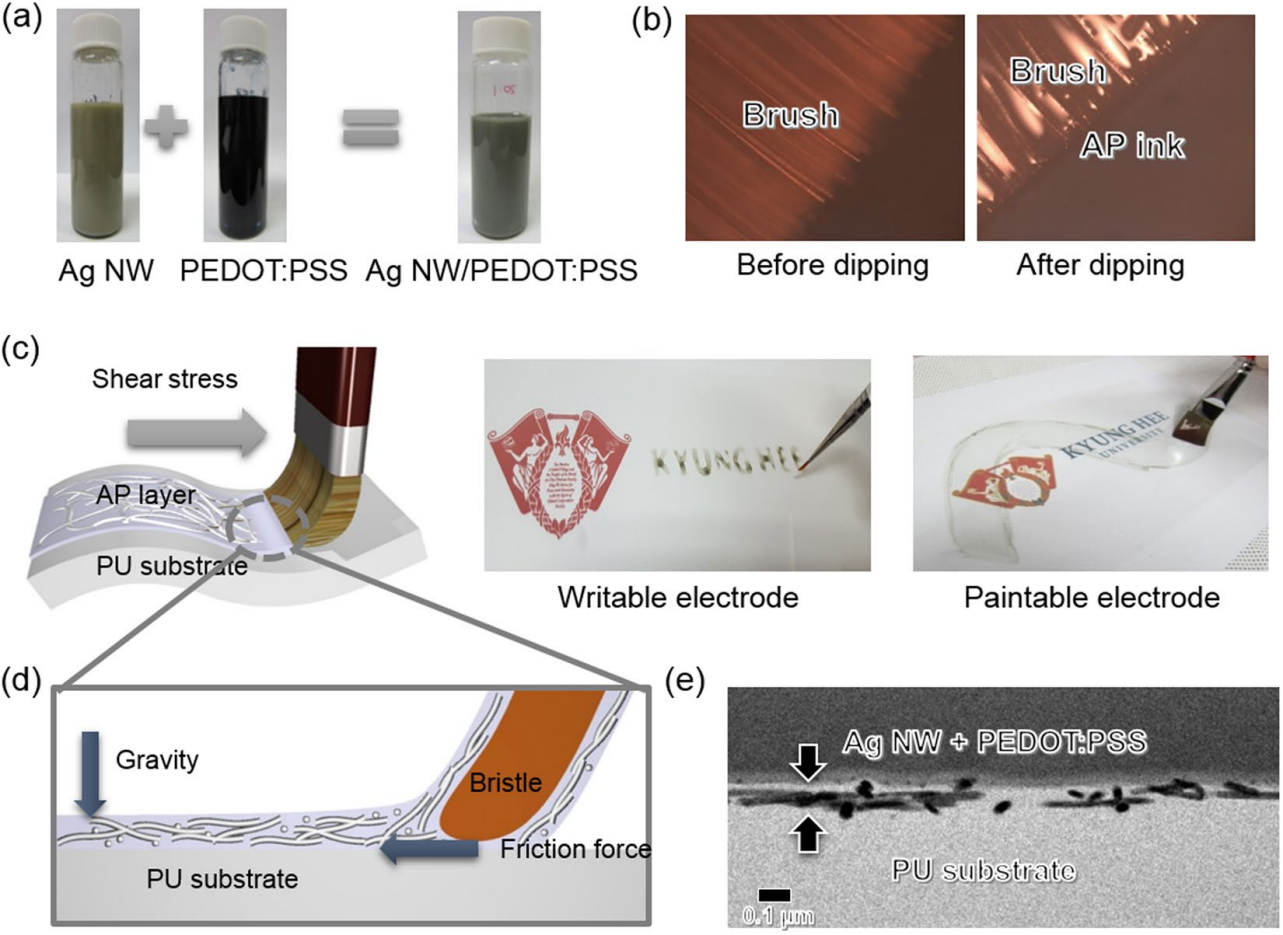
Preparation of a brush-painted stretchable electrode. (**a**) Ag nanowire and conducting PEDOT:PSS mixed ink (20 ml:1 ml). (**b**) Optical micrographs of a brush before and after dipping in the mixed Ag NW/PEDOT:PSS (AP) ink. (**c**) Schematic illustration of the brush painting process. Pictures show the writable and paintable electrode made by simple brush painting using different types of paintbrushes. (**d**) Brush painting process using mixed ink for laterally aligned Ag NWs in a PEDOT:PSS matrix. (**e**) Cross-sectional TEM image of a brush-painted stretchable hybrid electrode on PU substrate. The arrow indicates the electrode region on PU substrate. Brush process in (**c**) and (**d**) was drawn by using a RHINO drawing program.

To fabricate brush paintable and stretchable electrodes on a stretchable PU substrate, we prepared Ag NW and PEDOT:PSS and Ag NW mixed ink (20 ml:1 ml) as shown in Fig. 1a. The liquid ink used in our brush painting is a thick mixture of water-based PEDOT:PSS and Ag NWs. Although conductive PEDOT:PSS ink was employed, the brush-painted PEDDOT:PSS layer showed a high sheet resistance of 4.67 × 10^3^ Ohm/square. To improve the brush coatability of the mixed ink during the writing or painting process, we optimized the mixing ratio of solution PEDOT:PSS and Ag NW ink (Supporting Information). We used a general paintbrush made of nylon fibrils for brush painting^32–37^. Figure 1b shows optical microscopy (OM) images of paintbrush bristles before and after dipping into the Ag NW/PEDOT:PSS mixed ink. We observed that the Ag NW/PEDOT:PSS mixed ink was well absorbed on the bristles of the paintbrush. Using the optimized Ag NW/PEDOT:PSS ink and a regular paintbrush, we wrote or painted the Ag NW/PEDOT:PSS hybrid electrode on the stretchable PU substrates, which were attached on a mount heated to a temperature of 70 °C at a speed of 3.5 cm/s (see Supporting Information). To obtain a uniform morphology with controlled thickness, optimization of brush painting conditions (such as mount temperature and speed) was necessary. Figure 1c shows a schematic of the brush painting process using a narrow paintbrush for writing and a flat paintbrush for painting. As we discussed in our previous works, the shear stress of the paintbrush led to lateral alignment of the Ag NWs and the formation of well-connected Ag NW networks^29,32^. As discussed by Lee, the movement of the bristles is guided by their moment of inertia, the repulsive force from the substrate, and the external force on the brush applied by the person holding it^39^. The bristles absorbed conducting ink and left a trace on the PU substrate, as shown in Fig. 1c. In addition, the shear stress of the bristles resulted in a uniformly coated conducting PEDOT:PSS layer, which acts as a conducting matrix for the Ag NW network. As illustrated in Fig. 1d, we fabricated fairly thin paintable electrodes on PU substrates because the bristle of the paintbrush directly contacted the PU substrate, unlike conventional bar-coating or slot die coating^29,31^. The combination of the shear stress of the bristles and the friction force on the PU substrate led to very thin brush-painted Ag NW/PEDOT:PSS electrodes. Cross-sectional TEM images in Fig. 1e demonstrated that the Ag NWs in the PEDOT:PSS matrix were laterally aligned parallel to the PU substrate due to the shear stress of the paintbrush. Lateral alignment of the Ag NWs results in spike-free films, which provide excellent electrode reliability.

**Figure 2:**
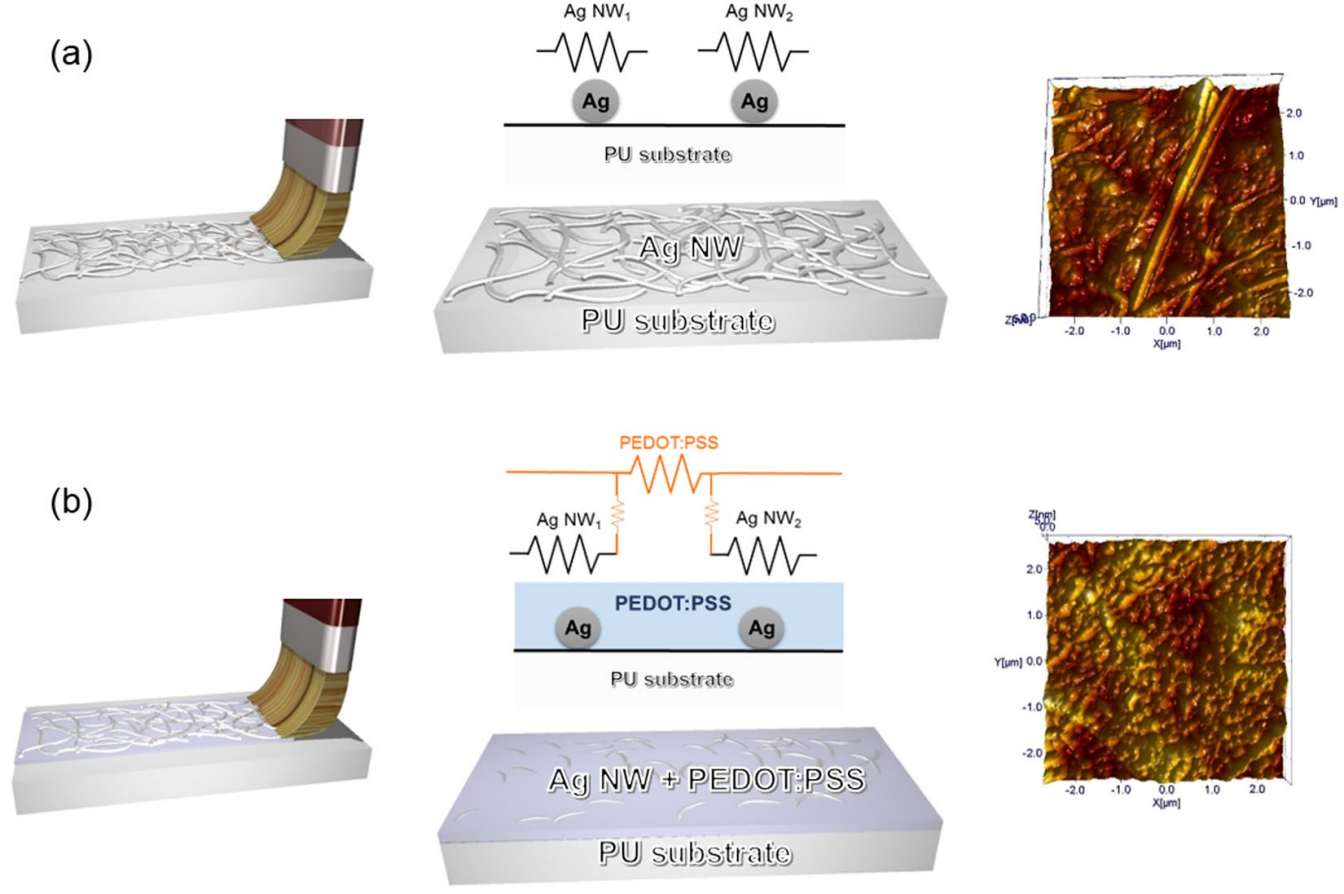
Schematic illustration of equivalent resistance circuit diagram and surface AFM images of brush-painted (**a**) Ag NW electrode and (**b**) Ag NW/PEDOT:PSS electrode on stretchable PU substrate. Brush process and schematic structure in (**a**) and (**b**) was drawn by using a RHINO drawing program.

Figure 2 shows the effect of conductive PEDOT:PSS in brush-painted Ag NW/PEDOT:PSS electrodes. In the brush-painted Ag NW network electrode without PEDOT:PSS (Fig. 2a), some Ag NWs were only weakly connected or were disconnected even though they were coated with paint. The brush-painted Ag NW electrode showed a fairly high sheet resistance due to existence of disconnected Ag NWs. The higher sheet resistance of the brush-painted Ag NW network can be understood using the equivalent resistance circuit diagram shown in Fig. 2a. In addition, a brush-painted Ag NW network on PU substrate has critical problems such as poor adhesion and irregular morphology. In stretchable electrodes, poor adhesion of the brush-painted Ag NW network electrode resulted in poor reliability and delamination of the Ag NW network from the PU substrate during stretching. However, brush-painted Ag NW/PEDOT:PSS electrodes showed a decreased sheet resistance of 19.7 Ohm/square due to the bridge effect of the conducting PEDOT:PSS between disconnected Ag NWs. The schematics and equivalent resistance circuit in Fig. 2b show the bridge effect of the conductive PEDOT:PSS layer. Brush-painted Ag NW/PEDOT:PSS electrodes showed a lower sheet resistance than brush-painted Ag NW electrodes because the disconnected Ag NWs were connected by conductive PEDOT:PSS. Chen *et al*. investigated Ag NW and graphene hybrid electrode and reported that the bridge effect of the 1-dimensional Ag NW on the 2-dimensional graphene sheet reduced the sheet resistance by effectively connecting the high resistance grain boundaries in graphene^40^. In addition, as discussed by Kim *et al*., ionic PEDOT chains interconnecting Ag NWs improved the electrical conductivity of brush-painted Ag NW/PEDOT:PSS hybrid films on PU substrate^26^. Furthermore, the surface morphology of the brush-painted Ag NW significantly improved because all Ag NWs were embedded in the conductive PEDOT:PSS layer. As shown in the surface AFM images in Fig. 2a and b, the brush-painted Ag NW/PEDOT:PSS electrode had a smoother surface and lower RMS roughness (16.9 nm) than the brush-painted Ag NW electrode (31.3 nm).

**Figure 3:**
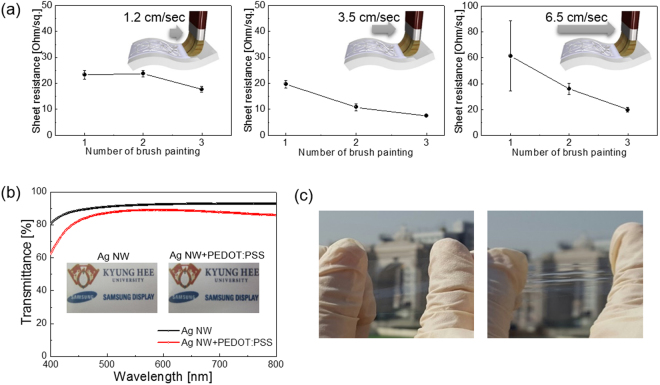
(**a**) Sheet resistance of Ag NW/PEDOT:PSS hybrid electrode with increasing brush painting cycles as a function of brush painting speed. Brush process in insets was drawn by using a RHINO drawing program. (**b**) Optical transmittance of brush-painted Ag NW and Ag NW/PEDOT:PSS hybrid electrodes in a visible wavelength region. (**c**) Optical transparency of a brush-painted Ag NW/PEDOT:PSS hybrid electrode before and after PU substrate stretching.

Figure 3a shows the sheet resistance of the brush-painted Ag NW/PEDOT:PSS electrode on PU substrate as a function of the number of brush painting cycles and brush painting speed. Regardless of brush speed, an increase in the number of brush painting cycles led to a decrease in sheet resistance due to an increase in Ag NWs density. At a low brush painting speed (1.2 cm/sec), an increase in brush painting cycles led to a small decrease in sheet resistance from 23.33 Ohm/square to 17.73 Ohm/square. It is difficult to make uniformly painted electrodes at a low brush painting speed due to the slow drying of the solvent and difficulty in controlling the brush painting trace. At a brush painting speed of 3.5 cm/sec, an increase in the number of brush painting cycles significantly reduced the sheet resistance of the Ag NW/PEDOT:PSS hybrid electrode to 7.5 Ohm/square. A further increase in brush painting speed increased the sheet resistance again because there was not enough Ag NW/PEDOT:PSS ink on the PU substrate. Therefore, optimization of brush painting speed is necessary to obtain uniformly coated Ag NW/PEDOT:PSS electrodes with a low sheet resistance. Figure 3b shows the optical transmittance of the brush-painted Ag NW and optimized Ag NW/PEDOT:PSS hybrid electrode on PU substrate. Although the brush-painted Ag NW electrode showed a slightly higher optical transmittance, the brush-painted Ag NW/PEDOT:PSS hybrid electrode showed a high optical transmittance in the visible wavelength region, which is acceptable for the fabrication of transparent interconnectors and transparent thin film heaters. At a wavelength of 550 nm, the brush-painted Ag NW/PEDOT:PSS electrode showed a high optical transmittance of 88.64%. The picture in Fig. 3c demonstrates the transparency and color of the brush-painted Ag NW/PEDOT:PSS electrode before and after sample stretching. Our brush-painted Ag NW/PEDOT:PSS hybrid electrode maintained a high optical transparency even while being stretched.

**Figure 4:**
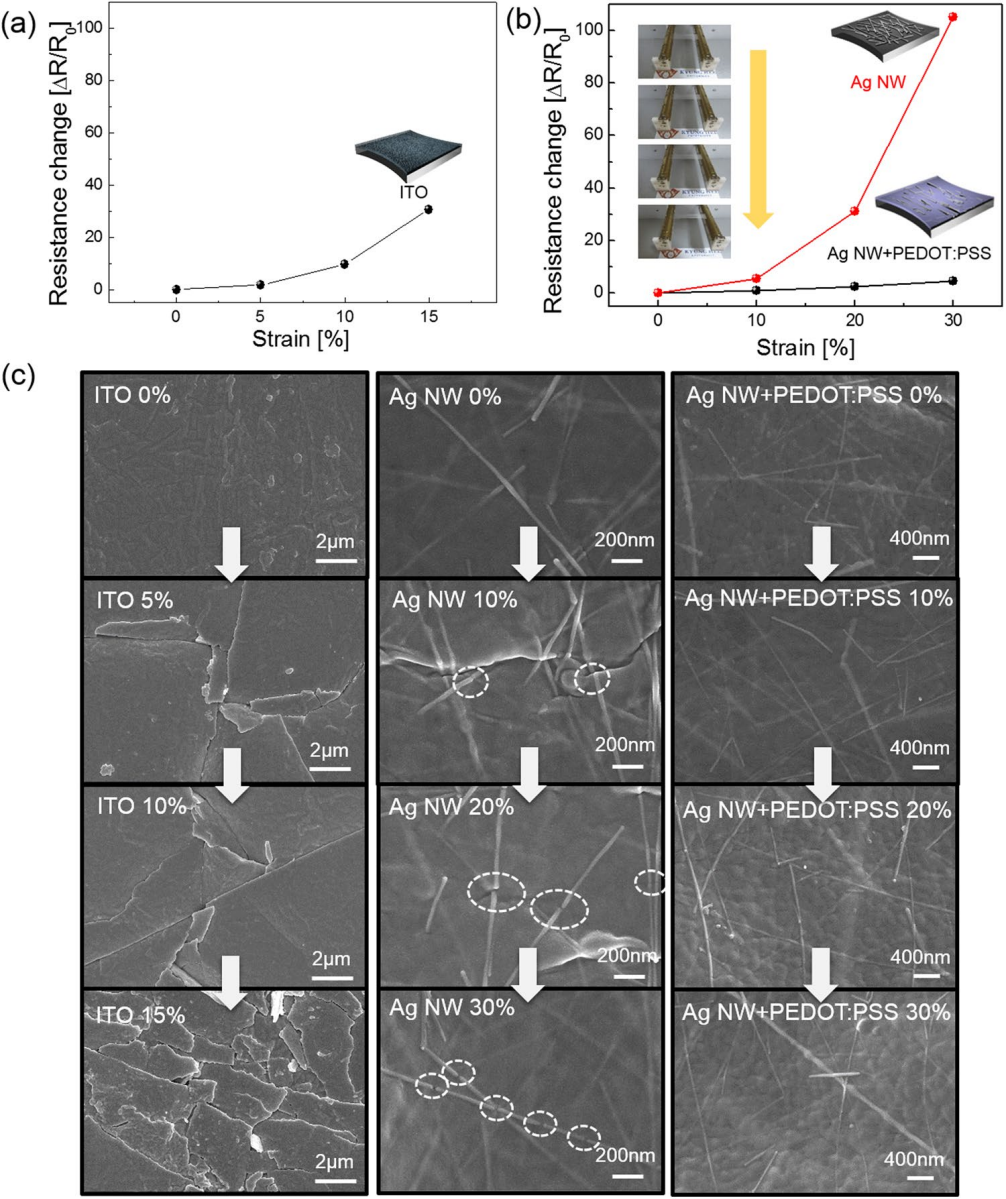
Resistance change of (**a**) sputtered ITO electrode on PU substrate and (**b**) brush-painted Ag NW and Ag NW/PEDOT:PSS electrode on PU substrate during stretching. The inset pictures show the stretching steps of the optimized Ag NW/PEDOT:PSS/PU sample. Insets of (**a**) and (**b**) were drawn by using a RHINO drawing program. (**c**) Surface FESEM images of sputtered ITO, brush-painted Ag NW, and Ag NW/PEDOT:PSS hybrid electrode before and after sample stretching.

Figure 4a shows the resistance change (ΔR/R_0_) of a DC sputtered ITO film with a thickness of 100 nm on a PU substrate during stretching. Due to brittleness of the sputtered ITO film, the sample showed an abrupt increase in resistance even with a strain of 15%. Figure 4b also shows a resistance change in the brush-painted Ag NW and Ag NW/PEDOT:PSS hybrid electrodes when the samples were strained from 0 to 30%. The inset pictures show the stretching steps of the sample using a specially designed stretching test system. Table 1 summarizes the sheet resistance change of brush-painted Ag NWs and Ag NW/PEDOT:PSS hybrid electrodes. Brush-painted Ag NW electrodes showed a significant increase in resistance change. The stretched Ag NW electrode showed a large resistance change at 30% strain due to its low initial sheet resistance (R_0_). However, the brush-painted Ag NW/PEDOT:PSS electrode maintained its initial sheet resistance even at a strain of 30%. Within strain of 20%, the stretching direction (parallel or orthogonal) of sample to the brush painting direction does not affect the resistance change. The high stretchability of brush-painted Ag NW/PEDOT:PSS hybrid electrodes was attributed to the existence of a highly stretchable PEDOT:PSS matrix. As expected from the cross sectional TEM image in Fig. 1e, the Ag NW embedded into the PEDOT:PSS matrix was easily stretched without a change in sheet resistance as the PEDOT:PSS matrix was stretched up to 30%^41^. Therefore, the Ag NW/PEDOT:PSS hybrid electrode maintained its sheet resistance even at a strain of 30%. Figure 4c showed surface FESEM images of the sputtered ITO, brush-painted Ag NW, and brush-painted Ag NW/PEDOT:PSS electrode with increasing strain. As expected from Fig. 4a, the increased sheet resistance of the ITO film on a PU substrate with increasing strain could be attributed to the generation and propagation of cracks perpendicular to the stretching direction. As discussed by Cairns *et al*., the ITO films sputtered on a flexible substrate showed severe cracks when the sample was bent due to the very small strain failure of 2.5%^42^. Therefore, even at a strain of 5%, the sputtered ITO film on PU substrate showed severe cracks. At a strain of 15%, the ITO film showed randomly propagated cracks, which separated the ITO film and increased the sheet resistance. The surface FESEM images of the brush-pained Ag NW also showed disconnected Ag NWs with increasing strain. The dashed circle in the FESEM images shows the disconnected Ag NWs region, which increased the sheet resistance of the brush-painted Ag NW electrode. In addition, the disconnected Ag NWs with short length could increase the sheet resistance when the substrate was severely stretched. However, the brush-painted Ag NW/PEDOT:PSS hybrid electrode showed similar surface FESEM images without cracks, delamination, or disconnected Ag NWs up to a strain of 30%. Similar surface FESEM images regardless of sample strain are consistent with a constant sheet resistance change of the stretched Ag NW/PEDOT:PSS electrode shown in Fig. 4b. In addition, we measured the contact angle of the bare PU, Ag NW/PU, and Ag NW/PEDOT:PSS/PU samples before and after stretching of 20 and 30% to observe the change of surface energy (Supporting Information). Compared to bare PU substrate with hydrophobic surface, the Ag NW and Ag NW/PEDOT:PSS showed a smaller contact angle due to high surface energy of the Ag NWs. After stretching of 20 and 30%, both Ag NW and Ag NW/PEDOT:PSS electrodes showed similar contact angle indicating a constant surface energy of Ag NW and Ag NW/PEDOT:PSS electrode after stretching. Consequently, the brush painted Ag NW/PEDOT:PSS hybrid electrode demonstrated comparable stretchability to the previously reported stretchable electrode such as CNT, Ag NW, PEDOT:PSS and transferred graphene sheet^7–10,21–23^.

**Table 1:** Sheet resistance change of the brush-painted Ag NW and Ag NW/PEDOT:PSS on PU substrates with increasing strain.

Strain [%]	Resistance change [ΔR/R_0_]	
	Ag NW	Ag NW + PEDOT:PSS
10%	5.551	0.84
20%	31.03	2.43
30%	105.19	4.71

**Figure 5:**
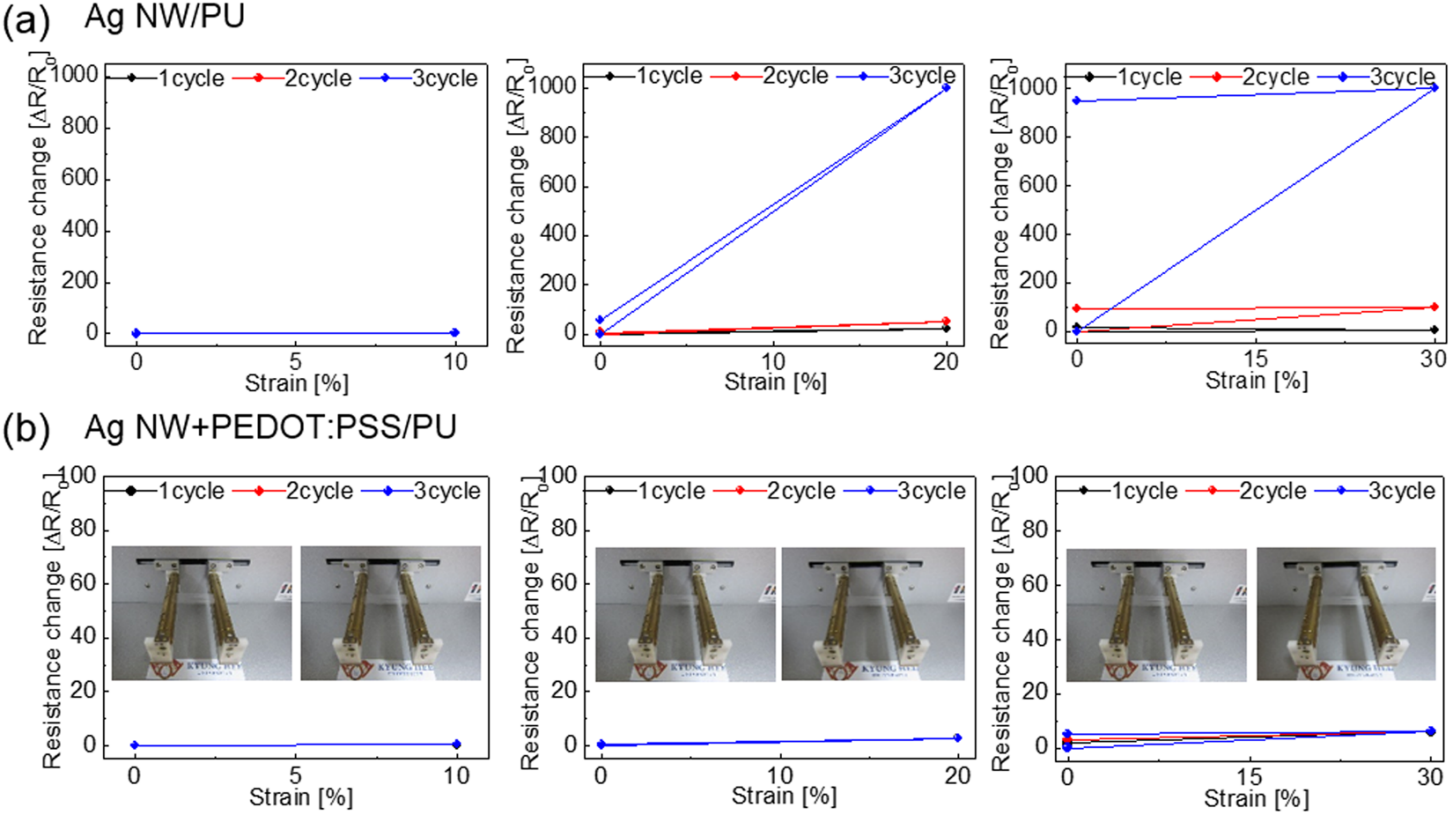
Hysteresis of resistance change of brush-painted (**a**) Ag NW and (**b**) Ag NW/PEDOT:PSS hybrid electrodes on PU substrate when samples were stretched from 10 to 30%. Inset pictures show stretching steps from 10 to 30%.

Figure 5 shows the hysteresis of resistance change for the brush-painted Ag NW and AgNW/PEDOT:PSS electrode as the substrates were stretched up to 30%. To compare the recovery properties of the brush-painted Ag NW and Ag NW/PEDOT:PSS on the PU substrates, we measured the resistance change of the electrodes before and after stretching from 10 to 30%. As shown in Fig. 5a, the brush-painted Ag NW/PU showed identical sheet resistance after 10% stretching. However, the 20% stretched Ag NW/PU sample showed significantly increased resistance after 3 cycles due to disconnected Ag NWs. The resistance change in the Ag NW/PU recovered somewhat after the strain was removed because physical contact was restored between disconnected Ag NWs. The 30% stretched Ag NW/PU sample showed an increased sheet resistance with increasing stretching cycles. Specifically, the sample that was stretched 3 times showed significantly increased sheet resistance even after removing strain. Once the severe cracks or disconnections formed on the Ag NWs during stretching, there was a permanent increase in the resistance of the Ag NW/PU sample even after the cracked Ag NW electrode returned to its original position. In contrast, the resistance of the brush-painted AgNW/PEDOT:PSS hybrid electrode on the PU substrate was fairly constant at 10 and 20% strain due to effective relaxation of the strain via the PEDOT:PSS matrix (Fig. 5b). When the strain was removed, the resistance of the Ag electrode returned to its initial value. Even at 30% strain, the brushed Ag NW/PEDOT:PSS electrode maintained its sheet resistance after removing the strain.

**Figure 6:**
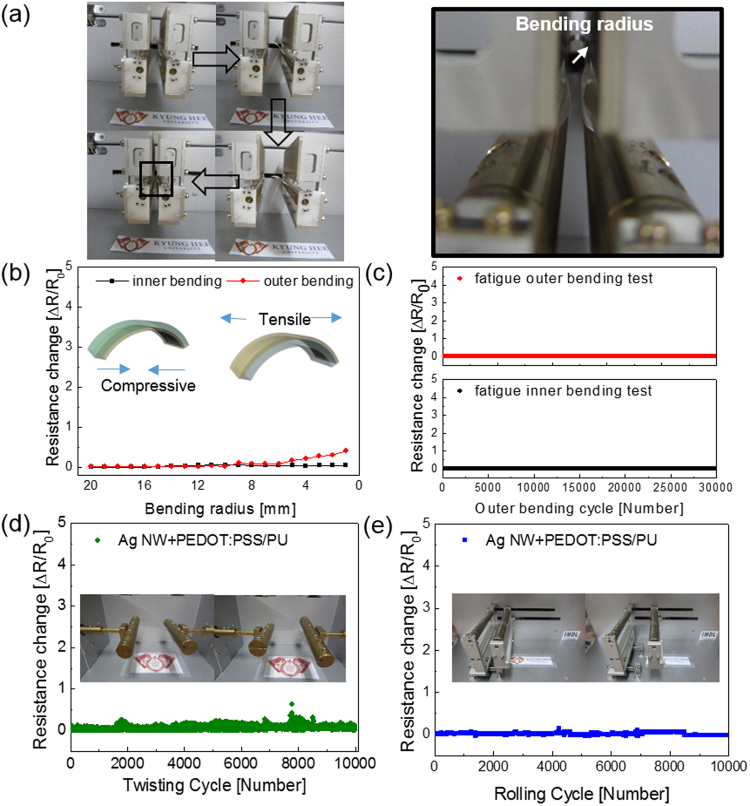
(**a**) Pictures show inner/outer bending of brush-painted Ag NW/PEDOT:PSS hybrid electrodes, and the enlarged picture shows the bending radius of the outer bended sample. (**b**) Resistance change of inner/outer bending of the brush-painted Ag NW/PEDOT:PSS sample with decreasing bending radius. Curved sample pictures in inset were drawn by using a RHINO drawing program. (**c**) Dynamic inner/outer bending fatigue test of a brush-painted Ag NW/PEDOT:PSS electrode at a fixed bending radius of 1 mm with repeated bending cycles (30,000 cycles). (**d**) Repeated twisting tests and (**e**) rolling tests of the brush-painted Ag NW/PEDOT:PSS electrode at a fixed twisting angle of 15 ° and rolling radius of 10 mm, respectively. Inset pictures show the twisting and rolling steps.

We measured resistance changes during outer and inner bending using a bending test system to investigate the mechanical flexibility of the optimized brush-painted Ag NW/PEDOT:PSS on PU substrate, which had a sheet resistance of 19.7 Ohm/square and an optical transmittance of 88.64% (Supporting Information). Figure 6a shows the results of outer/inner bending test steps with decreasing bending radius. The change in resistance of the brush-painted Ag NW/PEDOT:PSS electrode can be expressed as (ΔR = R − R_0_)/R_0_, where R_0_ is the initial measured resistance, and R is the *in-situ* measured resistance under substrate bending. The outer bended sample experienced tensile stress, while the inner bended sample film experienced compressive stress, as illustrated in the insets of Fig. 6b. The outer/inner bending test results showed that the brush-painted Ag NW/PEDOT:PSS electrode had a constant resistance change until a bending radius of 1 mm. This flexibility is sufficient for the fabrication of highly flexible interconnectors and thin films heaters for wearable and stretchable devices. Figure 6c shows the dynamic outer and inner bending fatigue test results obtained for the brush-painted Ag NW/PEDOT:PSS electrode with increasing repeated bending cycles (30,000 times) at a fixed bending radius of 1 mm. Both dynamic outer and inner bending fatigue tests of the brush-painted Ag NW/PEDOT:PSS hybrid electrode revealed no change in resistance after 30,000 bending cycles, indicating that the brush-painted Ag NW/PEDOT:PSS electrode had good flexibility and stability. Figure 6d shows the results of the dynamic twisting test of the brush-painted Ag NW/PEDOT:PSS with increasing twisting cycles at a fixed twisting angle of 15°. The inset pictures show twisting steps of the brush-painted Ag NW/PEDOT:PSS electrode. The ΔR/R_0_ value of the Ag NW/PEDOT:PSS electrode was almost unchanged with repeated twisting for 10,000 cycles. Figure 6e also shows the dynamic rolling test of the brush-painted Ag NW/PEDOT:PSS with repeated rolling cycles at a fixed radius of 10 mm. The inset pictures in Fig. 6e showed a dynamic rolling step. For repeated rolling cycles, there was no change in *in-situ* measured resistance due to the high flexibility of the Ag NW/PEDOT:PSS hybrid electrode.

**Figure 7:**
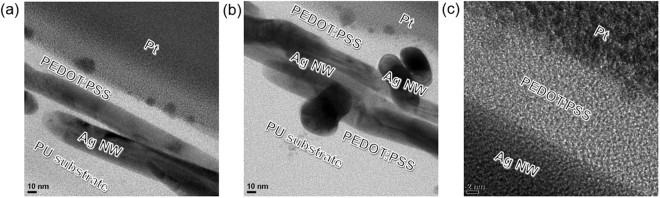
Enlarged cross sectional TEM images of (**a**) only parallel aligned Ag NWs and (**b**) perpendicularly aligned and parallel aligned Ag NWs embedded in a conductive PEDOT:PSS matrix. (**c**) Interface between Ag NW and PEDOT:PSS matrix prepared by brush painting.

High resolution transmittance electron microscopy (TEM) was employed to investigate the microstructure and interface structure of the brush-painted Ag NW/PEDOT:PSS hybrid electrode. Figure 7a shows a cross-sectional TEM image of the brush-painted Ag NW/PEPOT:PSS electrode on a PU substrate. As shown, parallel aligned Ag NWs were embedded in the conductive PEDOT:PSS matrix. The Ag NWs in the PEDOT:PSS matrix were parallel aligned to the PU substrate due to the shear stress applied by the paint brush. Some Ag NWs were perpendicularly connected to the Ag NWs, as shown in Fig. 7b. Regardless of Ag NW direction, all Ag NWs were uniformed embedded in the PEDOT:PSS matrix. As illustrated in Fig. 2b, the conductive PEDOT:PSS matrix acted as a bridge between disconnected Ag NWs and reduced the sheet resistance of the Ag NW/PEDOT:PSS electrode. In addition, the surface morphology of the PEDOT:PSS matrix followed the morphology of the Ag NW network, as shown in Fig. 7a and b. An enlarged cross sectional TEM image (Fig. 7c) shows a well-defined interface between crystalline Ag NWs and the amorphous PEDOT:PSS matrix. The Ag NWs were in good contact with the PEDOT:PSS without voids or defects, which allowed current to easily flow though this interface.

**Figure 8:**
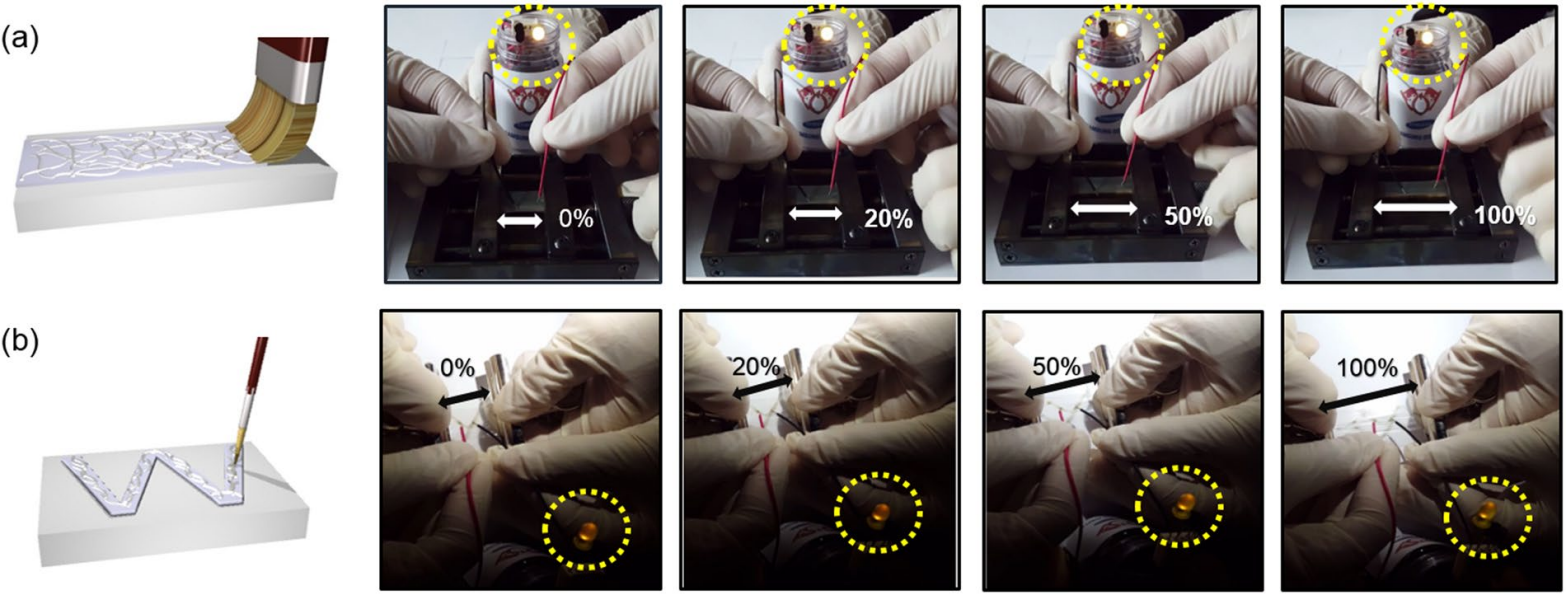
(**a**) Paintable and (**b**) writable Ag NW/PEDOT:PSS electrodes prepared using a flat paintbrush and small paintbrush, respectively, for use as stretchable and transparent interconnectors for LEDs. Continuous light emission up to a strain of 100% indicates successful operation as stretchable interconnectors. Panting and writing processes were drawn by using a RHINO drawing program.

Figure 8a shows the feasibility of paintable electrodes prepared using a flat paintbrush on stretchable PU substrate. The surface of the PU substrate was covered by Ag NW/PEDOT:PSS hybrid electrodes using a flat paintbrush, as illustrated in Fig. 8a. The paintable Ag NW/PEDOT:PSS hybrid electrode was connected to conventional white light emitting diodes and was slowly stretched. The LEDs remained on until the Ag NW/PEDOT:PSS hybrid electrode was stretched up to 100%, indicating that current flowed through the stretched Ag NW/PEDOT:PSS electrode up to this point. Writable Ag NW/PEDOT:PSS electrodes prepared using a small paintbrush also acted as stretchable electrodes, as shown in Fig. 8b. Even at a strain of 100%, the LED connected by the writable Ag NW/PEDOT:PSS electrode was turned on, indicating current flow through the writable electrode. These results imply that the fabricated stretchable interconnect is suitable for stretchable or wearable electronic devices and can be applied by a simple brush painting process onto any surface.

**Figure 9:**
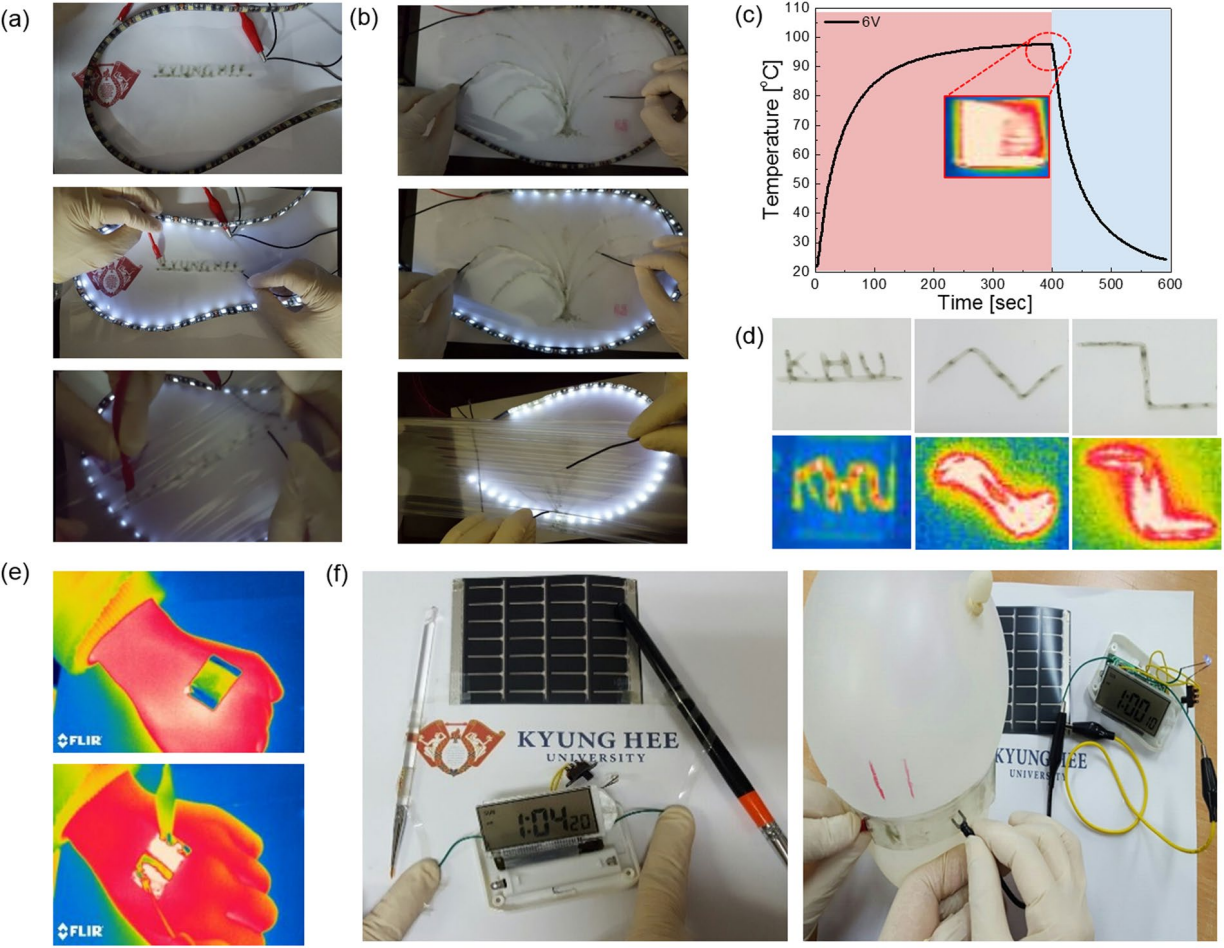
Promising applications of a brush-painted Ag NW/PEDOT:PSS hybrid electrode on PU substrate. (**a**) Pictures show that writable characters applied by brush painting act as interconnects for white LEDs. (**b**) Pictures show a brush-painted oriental orchid design on a PU substrate, which acts as an interconnector for LED belts. (**c**) Temperature profile of stretchable thin film heaters fabricated with a brush-painted Ag NW/PEDOT:PSS electrode. Inset shows an IR image of the stretchable and wearable thin film heater. (**d**) Pictures of writable Ag NW/PEDOT:PSS hybrid electrodes on PU substrate, and an IR image of the thin film heater fabricated on the writable electrodes. (**e**) IR image of stretchable thin film heaters with a brush-painted Ag NW/PEDOT:PSS electrode attached on the back of a person’s hand. (**f**) Brush-painted Ag NW/PEDOT:PSS electrode rolled on using a paintbrush with a radius of 2.5 mm and a wrapped balloon connected to a flexible Si solar cell and a liquid crystal display.

Figure 9 demonstrates the potential application of the brush-painted Ag NW/PEDOT:PSS films as flexible and stretchable electrodes. Figure 9a and b show characters and an oriental orchid design prepared by brush painting using AgNW and PEDOT:PSS mixed ink. Before and after severe stretching, the brush-painted Ag NW/PEDOT:PSS characters and picture act as interconnectors for operation of LED belts due to high connectivity through the Ag NW network embedded in a highly stretchable PEDOT:PSS matrix. The brush-painted Ag NW/PEDOT:PSS electrode was also used as an electrode for stretchable and transparent thin film heaters (TFHs). The stretchable TFHs were fabricated using a two-terminal Ag contact configuration (Supporting Information). A DC voltage was applied to the stretchable TFHs by a power supply through sputtered Ag contact electrodes at the film edge. The temperature profile of stretchable TFHs was measured using a thermocouple placed on the surface of the TFHs. Figure 9c shows the temperature profiles of the brush-painted Ag NW/PEDOT:PSS electrode-based TFHs at a constant input voltage of 6 V. When a DC input voltage of 6 V was supplied to the stretchable TFHs, a saturation temperature of 100 °C was instantly achieved due to the dynamic balance between Joule heating and convection. Figure 9d shows a brush-painted character and line to fabricate TFHs. The IR images obtained from TFHs correspond to the brush-painted character and lines. To demonstrate the feasibility of stretchable and wearable TFHs with brush-painted Ag NW/PEDOT:PSS hybrid electrodes, the TFHs were attached to the back of a person’s hand as shown in Fig. 9f. Changes in the IR image indicate that the TFH had a temperature of 46.1 °C when applying a voltage of 6 V. This stretchable TFH can be used as a wearable TFH to help the permeation of drugs through the skin for several types of patches. Brush-painted Ag NW/PEDOT:PSS electrodes can also be applied to simple interconnectors for portable devices. By brush painting and cutting of the PU substrate, we can simply prepare the interconnector instead of using a metal interconnect, which is difficult to cut in a special shape. Because the brush painting process is very simple, we can prepare any shaped transparent and stretchable interconnector with metallic conductivity. As shown in Fig. 9f, the brush-painted Ag NW/PEDOT:PSS electrode was used to connect flexible solar cells and LCDs. The operation of the LCD indicated the successful flow of the current generated by the solar cell through the Ag NW/PEDOT:PSS electrode painted with a small, flat brush. Due to the superior stretchability of the brush-painted Ag NW/PEDOT:PSS electrode, the stretchable electrode wrapped around the balloon was successfully operated as an electrode even with increasing balloon volume.

## Discussion

We demonstrated writable and paintable Ag NW/PEDOT:PSS hybrid electrodes on stretchable PU substrate using a conventional paintbrush. By direct brush painting of Ag NW/PEDOT:PSS mixed ink on heated PU substrate, we can obtain highly stretchable and transparent electrodes with a low sheet resistance of 19.7 Ohm/square, high optical transmittance of 88.64%, and high stretchability under ambient conditions. Shear stress-induced lateral alignment of Ag NWs into the conductive PEDOT:PSS matrix by brush painting led to a lower sheet resistance, outstanding stretchability, and stable hysteresis due to the bridge and reinforcement effects of the PEDOT:PSS matrix completely covering the Ag NWs. In addition, brush-painted Ag NW/PEDOT:PSS hybrid electrodes showed invariable resistance during inner/outer bending, stretching, and twisting tests, unlike conventional ITO films. The brush-painted Ag NW/PEDOT:PSS characters or pictures on PU substrate acted as good stretchable and transparent interconnectors and electrodes for stretchable electronics and wearable thin film heaters. The successful operation of writable and painted Ag NW/PEDOT:PSS hybrid films as stretchable interconnectors indicates the possibility of a simple brush painting process for paintable, stretchable, and wearable electronics.

## Methods

### Simple brush painting of Ag NW/PEDOT:PSS electrodes

Ag NWs (length 25 μm, width 30 nm, DI water 0.1%) and conductive PEDOT:PSS (Clevios HTL Solar) mixed ink (20 ml:1 ml) were used to brush paint Ag NW/PEDOT:PSS hybrid electrodes on stretchable PU substrates (50 μm) that were attached to a 70 °C heated substrate mount by vacuum suction. Typical flat, small paintbrushes made of nylon fibrils were used to fabricate paintable and writable transparent electrodes. The electrical and optical properties of brush-painted Ag NW/PEDOT:PSS hybrid electrodes were optimized with respect to brush painting speed. After brushing painting of the Ag NW/PEDOT:PSS electrodes, the samples were *in-situ* dried on a heated substrate at 70 °C for 10 min (Supporting Information).

### Characteristics of brush-painted Ag NW/PEDOT:PSS electrodes

The electrical and optical properties of the brush-painted Ag NW/PEDOT:PSS electrodes were examined using a four-point probe and a UV/visible spectrometer (UV 540, Unicam). Sheet resistance of the brush painted Ag NW/PEDOT:PSS electrodes were measured at room temperature by means of Hall effect measurement with van der Pauw geometry. The surface morphology of the samples was examined using atomic force microscopy (AFM). In addition, the microstructural and surface properties of the Ag NW/PEDOT:PSS electrodes were analyzed using FESEM and HRTEM. The mechanical integrity of the brush-painted Ag NW/PEDOT:PSS electrodes was evaluated using a lab-made inner/outer bending and stretching machine. In addition, dynamic fatigue stretching (bending/twisting/rolling) tests were performed using a lab-designed cyclic stretching test machine operated at a frequency of 1 Hz.

### Evaluations of the stretchable interconnectors and thin film heaters

To demonstrate the feasibility of brush-painted Ag NW/PEDOT:PSS hybrid films as stretchable interconnectors, we connected brush-painted Ag NW/PEDOT:PSS interconnectors with commercial LEDs. In addition, the characters or pictures prepared by brush painting were directly connected to the LED belt to show the current flow during sample stretching. Furthermore, the brush-painted Ag NW/PEDOT:PSS electrode was connected to solar cells and liquid crystal displays. Conventional TFHs with two-terminal side contacts were fabricated on the brush-painted Ag NW/PEDOT:PSS hybrid electrodes. A 100-nm-thick Ag side contact electrode was sputtered onto the brush-painted electrode. A DC voltage was supplied to the brush-painted electrode by a power supply (OPS 3010, ODA Technologies) through a Ag contact electrode at the film edge. The temperature of the TFHs was measured using a thermocouple mounted on the surfaces of the TFHs and an IR thermal imager.

## Electronic supplementary material

Supplementary Information
