# Supplementary material for: Brush-paintable and highly stretchable Ag nanowire and PEDOT:PSS hybrid electrodes
Source: Sci Rep. 2017 Oct 31;7:14685. doi: 10.1038/s41598-017-14951-3 (PMC5665903; doi:10.1038/s41598-017-14951-3)
Supplement: Supplementary file 1 — Supplementary Information [file 41598_2017_14951_MOESM1_ESM.pdf]

Supporting Information

Brush-paintable and highly stretchable Ag nanowire and PEDOT:PSS hybrid electrodes

Ji-Eun Lim[a], *Sang-Mok Lee*[a], *Seok-Soon Kim*[b], *Tae-Woong Kim*[c], *Hyun-Woo Koo*[c], *and Han-Ki Kim*[a,]*

[a]Department of Advanced Materials Engineering for Information and Electronics, Kyung-Hee University, 1 Seochen-dong, Yongin-si, Gyeonggi-do 446-701, Republic of Korea
[b]Department of Nano and Chemical Engineering, Kunsan National University, Kunsan, Jeollabuk-do 753, Korea
[c]OLED R&D Center, Samsung Display, 230 Nongseo-dong, Yongin-si, Gyeonggi-do, 17113, Republic of Korea

*Corresponding author. E-mail address: imdlhkkim@khu.ac.kr

Fabrication of Ag NW and PEDOT:PSS mixed ink (AP ink). We employed commercial Ag NW ink (length 25 μm, width 30 nm, D.I water 0.1%) and PEDOT:PSS (Clevios HTL Solar) ink to prepare the Ag NW and PEDOT:PSS mixed ink for brush painting. To obtain a brush-painted hybrid electrode with good coatability and low sheet resistance, we optimized the Ag NW/PEDOT:PSS ratio in the mixed ink. Figure S1a shows the change in measured sheet resistance of brush-painted Ag NW/PEDOT:PSS electrodes. As shown, the sheet resistance of the brush-painted hybrid electrode was mainly affected by the content of Ag NW ink. The sheet resistance of the brush-painted Ag NW/PEDOT:PSS electrode was significantly reduced with increasing content of Ag NW ink in the mixed ink. However, considering the uniformity and coatability of the brush-painted electrode, a further increase in Ag NW ink content was not desirable because the viscosity of the Ag NW/PEDOT:PSS mixed ink critically depends on the content of PEDOT:PSS ink. Therefore, the optimized mixing ratio of Ag NW and PEDOT:PSS ink was 20 ml: 1 ml. We obtained an optimized mixed ink for brush painting by stirring the 1 ml PEDOT:PSS and 20 ml Ag NW ink for 24 hours, as shown in Figure 1Sb.

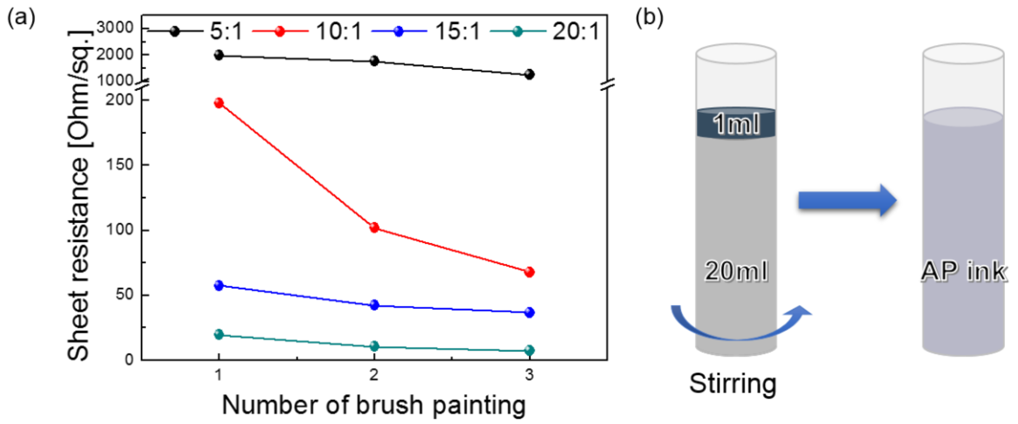

Figure S1. (a) Sheet resistance of a Ag NW/PEDOT:PSS hybrid electrode as a function of Ag NW and PEDOT:PSS mixing ratio. (b) Fabrication of Ag NW and PEDOT:PSS mixed ink for the brush painting process.

Brush painting process of Ag NW/ PEDOT:PSS mixed ink. The AP mixed ink was prepared at a mixing ratio of 20 ml:1 ml (Ag NW : PEDOT:PSS) by stirring for 24 hours. Then, the Ag NW and PEDOT:PSS hybrid films were fabricated by simple brush painting on a 70°C heated mount under atmospheric conditions, as shown in Figure S2a. The brush painting process was carried out on the heating mount of the conventional bar coating system. Brush painting of the Ag NW/PEDOT:PSS hybrid film was carried out on a stretchable PU substrate as shown in Figure S2b using a general paintbrush made of nylon fibrils. Because the optical transmittance of the PU substrate was affected by UV ozone treatment, the brush painting process was carried out without conventional UV ozone treatment. Therefore, the AP ink was directly painted on the PU substrate. After full dipping of the paintbrush into the AP ink, the AP layer was brushed on the PU substrate at a speed of 3.5 cm/s while heating the PU substrate on the heated mount. After brush painting, the AP layer was dried at 70°C for 10 min under ambient conditions to remove the solvent.

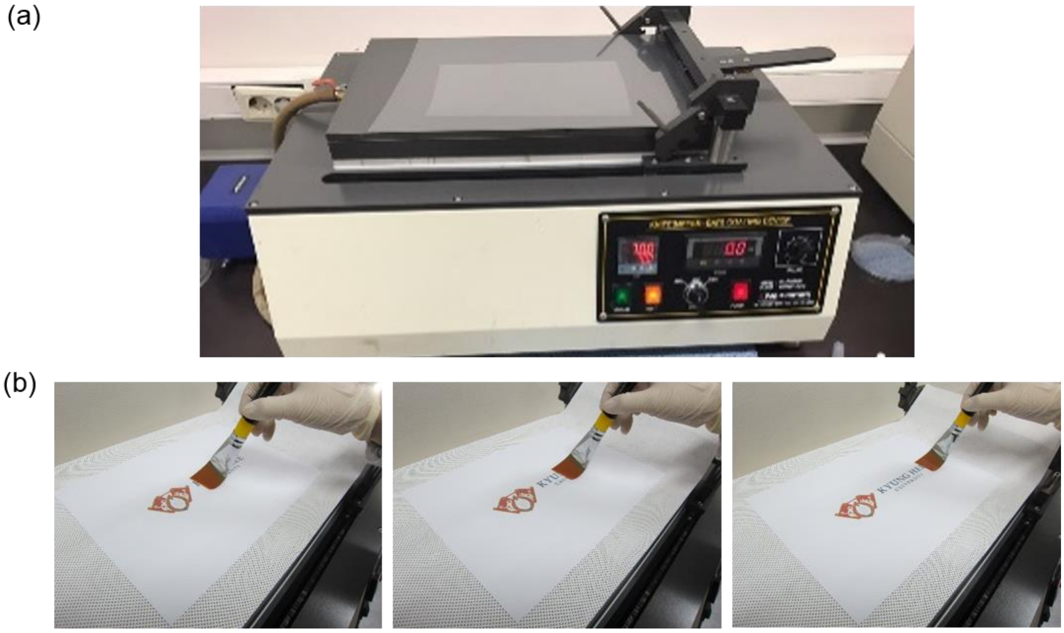

Figure S2. (a) Heated mount for brush painting and (b) brush painting process of Ag NW/PEDOT:PSS film on PU substrate using a typical flat paintbrush.

Stretching, bending, twisting, and rolling test of brush-painted Ag NW/PEDOT:PSS electrodes. The mechanical integrity of the brush-painted Ag NW/PEDOT:PSS electrode was evaluated using a lab-made stretching, bending, twisting, and rolling test machine as shown in Figure S3a-d. In the stretching hysteresis test, we measured the resistance change of the electrodes before and after stretching from 10 to 30% with increasing stretching cycles. The system can automatically measure the change of resistance of the brush-painted electrodes during the outer or inner bending tests as a function of bending radius. Brush-painted Ag NW/PEDOT:PSS films with a size of 1.5 x 5 cm^2 were tightly mechanically clamped between Cu plates. First, we measured the resistance change of the brush-painted Ag NW:PEDOT:PSS electrodes as a function of bending radius.

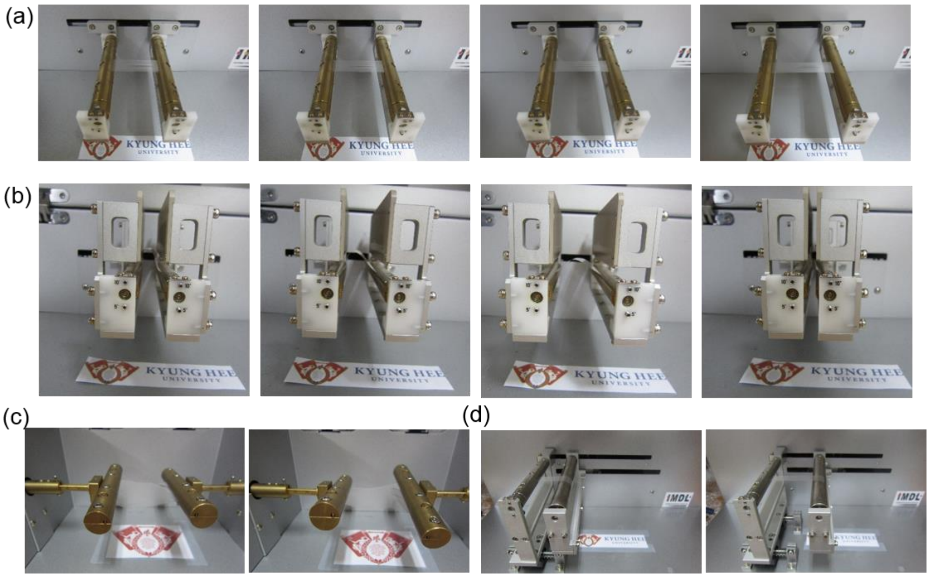

Figure S3. Lab-made bending test system to investigate the mechanical flexibility and stretchability of the brush-painted Ag NW/PEDOT:PSS electrodes. Specially designed a) stretching tester, b) inner/outer bending tester, c) twisting tester, and d) rolling tester used to measure the resistance change of the brush-painted Ag NW/PEDOT:PSS samples.

Secondly, we investigated dynamic fatigue bending by repeating the outer and inner bending mode. The bending radius and frequency were approximately 1 mm and 1 Hz, respectively

(Figure S3b). Figure S3c shows the steps in the dynamic twisting test of the brush-painted Ag NW/PEDOT:PSS with increasing twisting cycles at a fixed twisting angle of 15^0. Figure S3d also shows a dynamic rolling step with repeated rolling cycles at a fixed radius of 10 mm.

Measurement of contact angle on brush painted Ag NW/PEDOT:PSS electrode. To show the surface properties of the stretchable Ag NW and Ag NW/PEDOT:PSS electrodes, we measured and compared contact angle of water on the bare PU substrate, Ag NW/PU, and Ag NW:PEDOT:PSS/PU before and after strain of 20 and 30% as shown in Figure S4a.

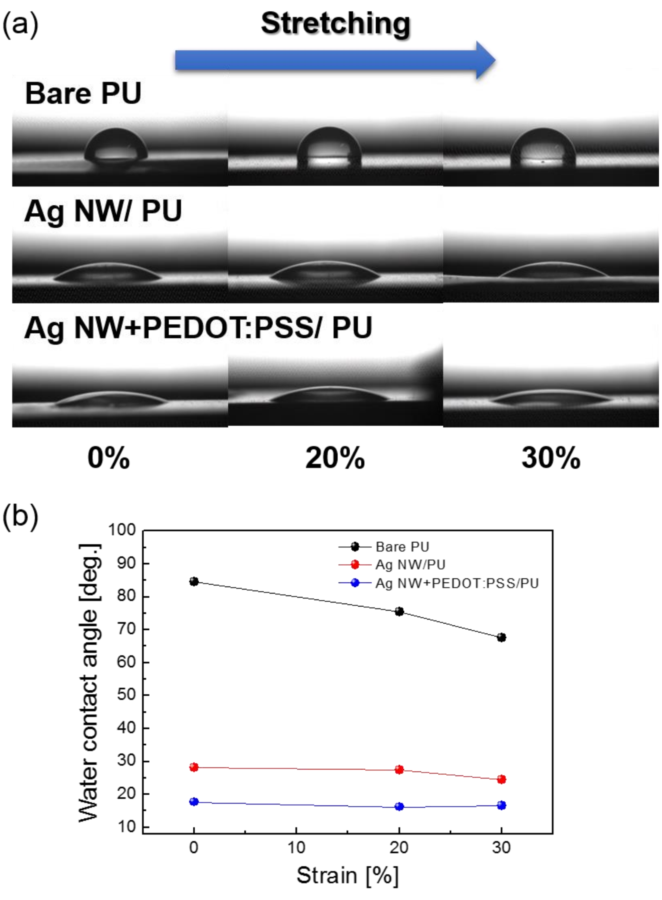

Figure S4. (a) Optical microscope images of the water droplet on bare PU substrate, Ag NW/PU, and Ag NW/PEDOT:PSS/PU samples. (b) Measured contact angle of bare PU substrate, Ag NW/PU, and Ag NW/PEDOT:PSS/PU samples before and after stretching of 20 and 30 %.

Compared to the contact angle of hydrophobic PU substrate, the Ag NW and Ag NW/PEDOT:PSS electrode showed much smaller contact angle due to high surface energy of the Ag NWs. Even after stretching of 20 and 30%, the Ag NW and Ag NW/PEDOT:PSS electrode showed similar contact angle as shown in Figure 4 Sb indicating constant surface energy of the brush pained Ag NW and Ag NW/PEDO:PSS electrodes.

Fabrication of stretchable TFHs. To demonstrate the feasibility of the brush-painted Ag NW/PEDOT:PSS hybrid electrode as a transparent electrode for stretchable TFHs, TFHs were fabricated as shown in Figures S5a and b. Two-terminal side Ag contacts were sputtered onto the edge of the brush-painted Ag NW/PEDOT:PSS hybrid electrode to apply power to the TFHs. A DC voltage was supplied by a power supply (OPS 3010, ODA Technologies) to the brush-painted Ag NW/PEDOT:PSS-based TFHs through an Ag contact electrode at the film edge. The temperature of the TFHs was measured using a thermocouple mounted on the surfaces of the TFHs and an IR thermal imager (A35sc, FLIR), as shown in Figure S4c.

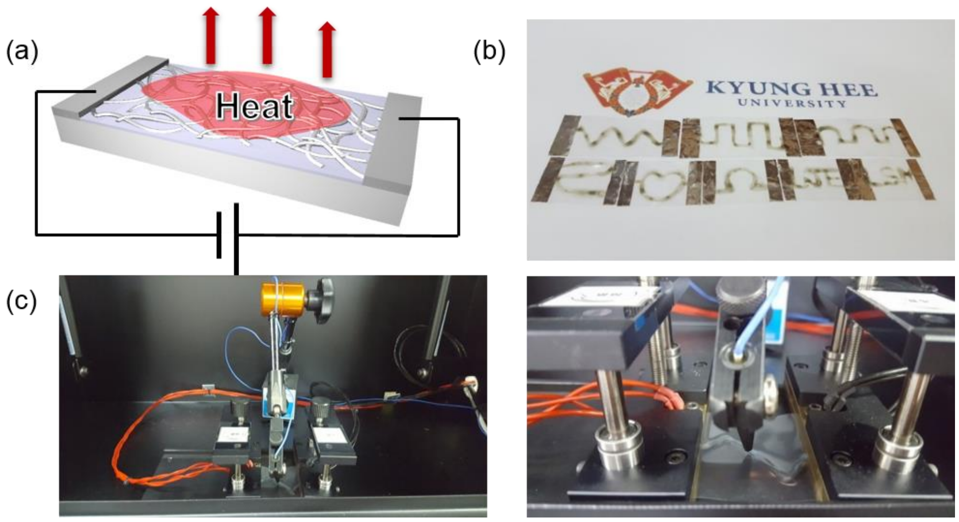

Figure S5. a) Schematic structure of a TFH fabricated on a brush-painted Ag NW/PEDOT:PSS electrode. Schematic structure was drawn by using a RHINO drawing program. b) Brush-painted Ag NW/PEDOT:PSS electrodes on PU substrate with a sputtered

Ag side contact. (c) Temperature measurement system for Ag NW/PEDOT:PSS electrode-based TFHs.
